# Monogenic lupus with *SLC7A7* mutations: a retrospective study from a Chinese center

**DOI:** 10.1186/s13023-026-04258-w

**Published:** 2026-03-13

**Authors:** Yifan Li, Qianying Lv, Wei Lu, Haimei Liu, Jiayan Feng, Wanzhen Guan, Yinv Gong, Qiaoqian Zeng, Xiaomei Zhang, Hong Xu, Li Sun

**Affiliations:** 1 Department of Rheumatology, Children’s Hospital of Fudan University, National Center for Children’s Health, Shanghai, China; 2 Department of Pediatric Endocrinology and Inherited Metabolic Diseases, Children’s Hospital of Fudan University, National Center for Children’s Health, Shanghai, China; 3 Department of Pathology, Children’s Hospital of Fudan University, National Center for Children’s Health, Shanghai, China; 4 Department of Nephrology, Children’s Hospital of Fudan University, National Center for Children’s Health, Shanghai, China

**Keywords:** Lysinuric protein intolerance, Monogenic lupus, SLC7A7, Systemic lupus erythematosus

## Abstract

**Background:**

Monogenic lupus is a rare but severe form of systemic lupus erythematosus (SLE) that typically manifests during childhood. Mutations in *SLC7A7* cause lysinuric protein intolerance (LPI), and this gene has been implicated in monogenic lupus. This study aimed to investigate the clinical features, treatment strategies, and outcomes of pediatric patients with monogenic lupus caused by *SLC7A7* mutations.

**Methods:**

We conducted a retrospective single-center study of pediatric patients with SLE who fulfilled the 2012 SLICC and/or 2019 EULAR/ACR classification criteria and underwent next-generation sequencing because of early onset or atypical clinical features. Patients carrying biallelic pathogenic or likely pathogenic variants in *SLC7A7* together with biochemical evidence of LPI were classified as having *SLC7A7*-associated monogenic lupus. Their clinical and immunological characteristics, treatment strategies, and outcomes were compared with those of SLE patients without identifiable monogenic variants.

**Results:**

Among pediatric SLE patients who underwent next-generation sequencing for suspected monogenic etiology, six were identified with biallelic *SLC7A7* variants and biochemical evidence of lysinuric protein intolerance, accounting for 31.6% of all monogenic lupus cases in this cohort. The mean age at SLE diagnosis in the *SLC7A7*-associated monogenic lupus group was 6.6 years, and patients were followed for an average of 5.5 years. In the overall LPI cohort (*n* = 12), the median age of symptom onset was 1 year and the mean age at LPI diagnosis was 7.7 years. The recurrent splice-site mutation c.625 + 1G > A was the most frequent variant, detected in 14 of 24 alleles. Compared with gene-negative SLE patients, those with *SLC7A7*-associated monogenic lupus more frequently exhibited protein intolerance, osteopenia, short stature, and hyperferritinemia (all *p* < 0.05). Treatment consisted of combined immunosuppressive and metabolic therapies, and at the last follow-up 83.3% (5/6) of patients had achieved and maintained a Lupus Low Disease Activity State (LLDAS).

**Conclusions:**

In Chinese patients with lysinuric protein intolerance, this study suggests an association with an increased susceptibility to developing SLE. Given the small LPI-SLE subgroup, these findings require validation in larger cohorts. Within this cohort, the splice-site mutation c.625 + 1G > A was the most frequently observed *SLC7A7* variant. Furthermore, in children diagnosed with SLE, the presentation of a metabolic triad—protein intolerance, short stature, and osteopenia—should raise suspicion for *SLC7A7*-associated monogenic lupus and prompt genetic and metabolic evaluation, which could facilitate early diagnosis and timely intervention.

**Supplementary Information:**

The online version contains supplementary material available at 10.1186/s13023-026-04258-w.

## Introduction

Systemic lupus erythematosus (SLE) is a multisystem autoimmune disease characterized by various autoantibody profiles and complex clinical manifestations. Childhood-onset SLE (cSLE) accounts for approximately 4–20% of all SLE cases and generally presents with more severe symptoms compared to adult-onset SLE [1–3]. Monogenic lupus, caused by variants in single genes, is a rare but severe form of SLE that typically manifests during childhood. Although monogenic SLE is rare, it is increasingly recognized, pathogenic variants in genes associated with monogenic SLE are identified in approximately 3–10% of early-onset SLE cases [4–6]. Genes implicated in monogenic SLE include those associated with complement defects, defects in activation of the type I IFN pathway, defects in T and B cell tolerance, and certain metabolic disorders [7, 8].

The solute carrier family 7 amino acid transporter light chain, y + L system, member 7 (SLC7A7; OMIM *603593; GenBank RefSeq: NM_003982), has been identified as a metabolic gene involved in monogenic SLE. *SLC7A7* encodes the y + L amino acid transporter-1 (y+LAT1), which, together with 4F2hc (surface antigen 4F2 heavy chain), exports cationic amino acids (CAAs—lysine, arginine, and ornithine) in exchange for neutral amino acids and sodium across the basolateral membrane of epithelial cells in the small intestine and kidney [9, 10]. Mutations in the *SLC7A7* gene cause lysinuric protein intolerance (LPI, OMIM #222700), an autosomal recessive aminoaciduria first reported in 1965 [11]. LPI has been sporadically reported worldwide, with a higher prevalence in Finland, Italy, and Japan.

The common clinical manifestations of LPI include aversion to protein-rich foods, failure to thrive, hyperammonemia, hepatosplenomegaly, osteoporosis, alveolar proteinosis, and immunological disorders. Reported immunological disorders include hemophagocytic lymphohistiocytosis, systemic lupus erythematosus, idiopathic thrombocytopenic purpura, and autoimmune encephalitis [12–14]. In 1994, Parto and colleagues reported a case of kidney involvement in an LPI patient, where renal biopsy suggested immune-mediated glomerulonephritis, potentially diagnosable as SLE [14]. Since then, additional cases presenting with both LPI and SLE have been reported.

In China, only sporadic cases of LPI have been reported, mostly by individual specialties such as gastroenterology, respiratory medicine, neurology, and rheumatology, and these isolated reports describe only selected manifestations of the disease rather than its full clinical spectrum in Chinese patients [15–22]. In our previous study systematically screening disease-causing genes in children with suspected monogenic lupus at our center, *SLC7A7* emerged as the most frequent causative gene, suggesting that LPI represents an under-recognized metabolic etiology of pediatric SLE in this population [23]. Given China’s large population, limited awareness of LPI and the lack of routine metabolic and genetic evaluation in children with SLE are likely to contribute to underdiagnosis of *SLC7A7*-associated lupus, diagnostic delay, and suboptimal management. To date, however, no cohort studies have systematically characterized the clinical features and prognosis of patients with LPI complicated by SLE. Therefore, this study aimed to delineate the clinical heterogeneity, mutational spectrum, and treatment outcomes of pediatric *SLC7A7*-associated monogenic lupus in a single Chinese center, in order to facilitate earlier recognition and guide targeted clinical interventions.

## Methods

### Patients and data collection

From June 2016 to June 2024, 420 children with SLE were admitted to the Department of Rheumatology at Children’s Hospital of Fudan University, China. SLE was diagnosed according to the 2012 SLICC or 2019 EULAR/ACR classification criteria, and haemophagocytic lymphohistiocytosis (HLH) was diagnosed based on the HLH-2004 criteria [24]. Demographic characteristics, clinical manifestations, laboratory results, imaging findings, organ involvement, medications, and other therapies were collected at diagnosis and during follow-up. Follow-up visits were scheduled monthly or every three months during active disease and every six to twelve months during quiescent phases. Disease activity was assessed at each visit using the SLEDAI-2000 (SLEDAI-2 K), and treatment outcomes were evaluated according to attainment of Lupus Low Disease Activity State (LLDAS) [25, 26]. 

**Fig. 1:**
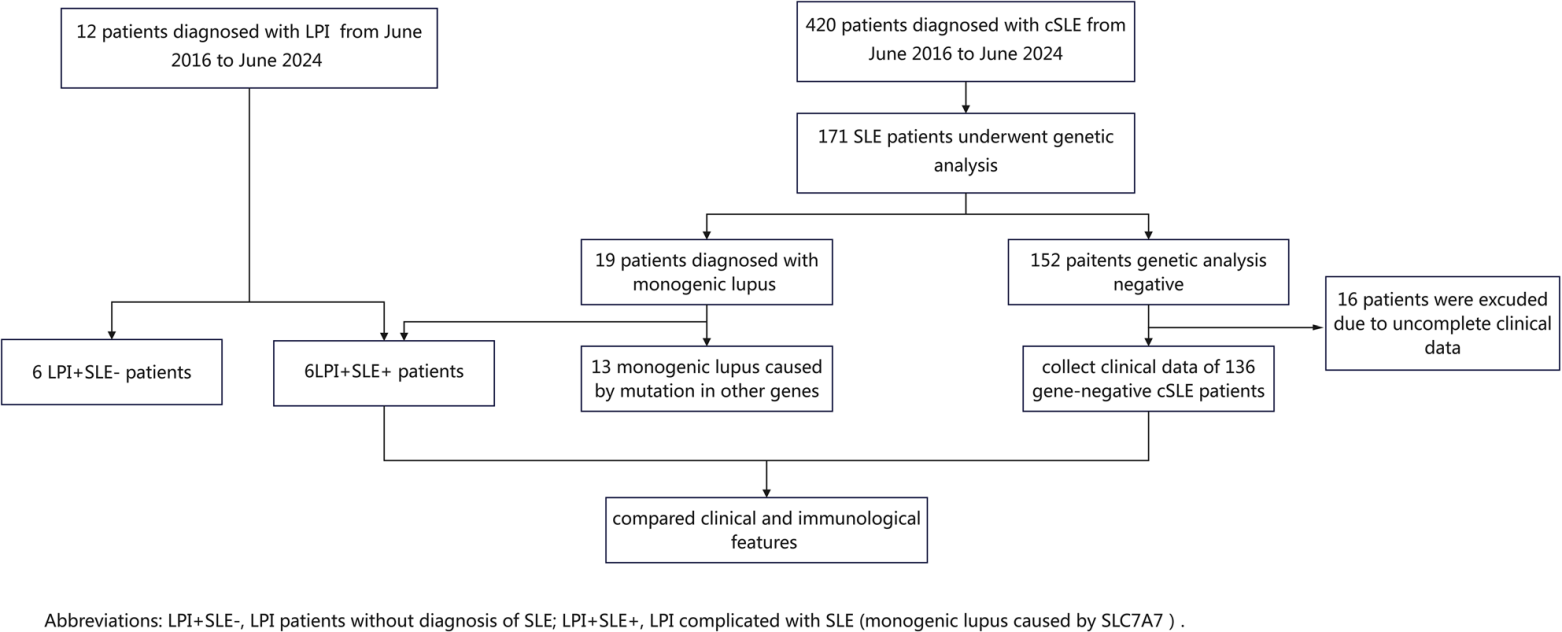
Patient enrollment and classification flowchart of the cohort

As illustrated in the flowchart (Fig. 1), 171 of the 420 SLE patients underwent genetic testing. Nineteen patients were identified as having monogenic lupus, while the remaining 152 had negative genetic results. Among the monogenic lupus cases, six carried homozygous or compound heterozygous *SLC7A7* variants and were classified as the *SLC7A7*-associated lupus group (LPI + SLE+), whereas the other thirteen harbored variants in genes unrelated to *SLC7A7*. After exclusion of sixteen SLE patients with incomplete clinical information, 136 genetically negative SLE patients were included as the gene-negative comparison group. Clinical and immunological characteristics were compared between the LPI + SLE+ group and the gene-negative SLE group.

In parallel, we retrospectively reviewed all twelve patients with genetically confirmed LPI seen at our center during the same period. All LPI patients carried homozygous or compound heterozygous mutations in *SLC7A7*. For this LPI cohort, we collected data on demographic features, clinical manifestations, laboratory parameters, imaging findings, organ involvement, and treatments to delineate the broader clinical spectrum of *SLC7A7* deficiency in Chinese children.

### Ethics

Written informed consent was obtained from all participants or their parents. This study was conducted in accordance with the principles of the Declaration of Helsinki and received approval from the Ethics Committee of Children’s Hospital of Fudan University (ethical approval number: No. [2023] 192).

### Statistical analysis

All statistical analyses were performed using SPSS software (version 23.0). The normality of continuous data was assessed using both the Shapiro-Wilk test and visual inspection of Q-Q plots. Based on this assessment, normally distributed data are presented as mean ± standard deviation (SD) and were compared using the Independent Samples T-test. Non-normally distributed data are presented as median with interquartile range (IQR) and were compared using the Mann-Whitney U test. Categorical variables are expressed as number of cases and percentage (n, %) and were analyzed using the Chi-square test or Fisher’s exact test, as appropriate. A two-sided p-value of less than 0.05 was considered statistically significant.

A post-hoc power analysis was performed using G*Power software (version 3.1) based on the observed effect sizes for key clinical comparisons, given the sample sizes of the study groups.

## Result

### General clinical spectrum of LPI patients

This study enrolled twelve LPI patients from nine unrelated families of Han Chinese ethnicity. The cohort consisted of five males and seven females, with one patient born to a third-degree consanguineous marriage. The median age at symptom onset was 1 year (IQR: 0.4, 2.5 years), with a marked diagnostic delay reflected by a median age at LPI diagnosis of 8.0 years (IQR: 6, 10.3 years). For the subset of patients who developed SLE (*n* = 6), the median age at SLE diagnosis was 11.5 years (IQR: 9.3, 13.8 years). Patients were followed for a mean duration of 5.5 years (SD: 3.4 years).

All patients exhibited the classic metabolic hallmarks of LPI. Protein intolerance and hyperammonemia were universal (12/12, 100%), accompanied by vomiting and diarrhea in 4 of 12 (33.3%) and 3 of 12 (25.0%) patients, respectively. Hyperferritinemia was also present in all patients (12/12). Among the 11 patients who underwent bone mineral density assessment, osteopenia was present in all patients (11/11, 100%). Osteoporosis was identified in three of these patients (3/11, 27.3%). Hepatosplenomegaly was noted in 10 patients (83.3%), and one patient (Case 1) had pancreatitis.

Hematological abnormalities were observed in 8 of 12 patients (66.7%), including anemia (8/12, 66.7%), leukopenia (2/12, 16.7%), and thrombocytopenia (3/12, 25.0%). Renal involvement was universal (12/12, 100%), primarily manifesting as renal tubulopathy, defined by elevated urinary α1-microglobulin and/or N-acetyl-beta-D-glucosaminidase (NAG). Proteinuria and microscopic hematuria were present in 5 (41.7%) and 2 (16.7%) patients, respectively. Notably, all patients maintained normal renal function at the last follow-up. Pulmonary complications occurred in five patients (41.7%), including interstitial lung disease (3/12) and alveolar proteinosis (2/12).

Immunological dysregulation was a key feature, affecting 7 patients (58.3%). Six patients were diagnosed with SLE (constituting the LPI + SLE+ group for subsequent analysis), and two of these six also fulfilled the criteria for HLH. In addition, one patient without SLE was diagnosed with HLH alone. The detailed clinical characteristics of the LPI cohort are summarized in Table S1.

### Genetic spectrum of the LPI cohort and the predominance of *SLC7A7* in monogenic lupus

**Fig. 2:**
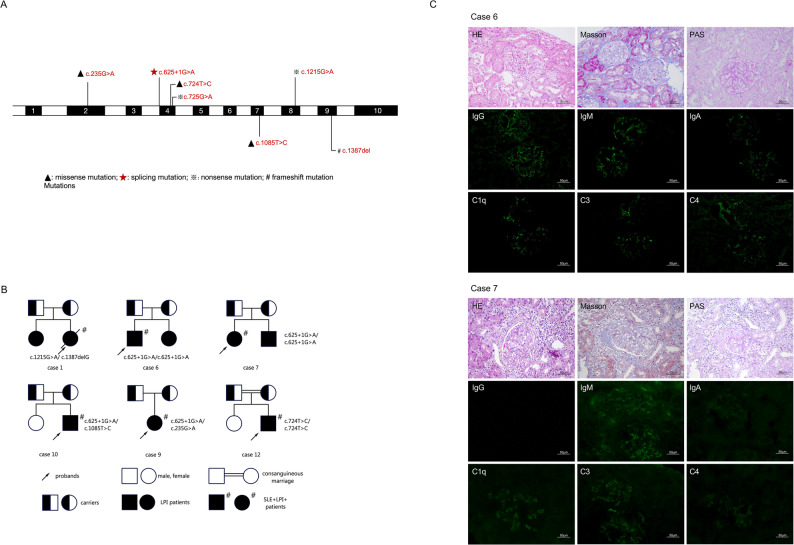
Genetic mutations, pedigree, and renal pathology of *SLC7A7*-associated disease. (**A**) The positions and frequencies of *SLC7A7* mutations identified in patients with LPI in our cohort. (**B**) Pedigrees of families with *SLC7A7*-associated monogenic lupus in the cohort, with individual genotypes and alleles (including the recurrent c.625 + 1G > A variant) indicated. (**C**) Renal biopsy pathology findings for Case 6 and Case 7. Case 6: Renal biopsy reveals mild segmental mesangial and matrix proliferation in a few glomeruli. Some glomerular capillary loops show adhesion to the Bowman’s capsule wall. Light microscopy identifies swelling and vacuolar degeneration of proximal tubular epithelial cells, protein casts, and cellular casts, alongside mild interstitial lymphocytic infiltration. Immunofluorescence microscopy shows IgG (+), IgA (+), IgM (+), C3 (+), C4 (±), C1q (++), Fb (±), C3d (+), and C9 (+). Case 7: Renal biopsy shows that half of the glomeruli exhibit mild segmental mesangial and matrix proliferation, with two glomeruli forming cellular fibrous crescents. Some glomerular capillary loops adhere to the Bowman’s capsule wall. Light microscopy demonstrates vacuolar degeneration of proximal tubular epithelial cells, focal necrosis in individual tubules with lymphocytic infiltration, and mild interstitial fibrosis with extensive lymphocyte and plasma cell infiltration. Immunofluorescence microscopy shows IgG (-), IgA (+++), IgM (±), C3 (+~++), C4 (±), C1q (+), and Fb (±)

Genetic analysis of the twelve patients identified eight distinct pathogenic mutations in the *SLC7A7* gene, including five point mutations (p.W242X, p.W242R, p.L362P, p.G79R, and p.W405X), one splicing mutation (c.625 + 1G > A), and one frameshift mutation (p.V463Cfs*56) (Table 1). The splice-site mutation c.625 + 1G > A was the most prevalent, found in a homozygous state in four non-consanguineous families and in a heterozygous state in two others, accounting for 14 of the 24 total alleles (Fig. 2A).

**Table 1:** Detailed clinical and immunological profiles of the six patients with *SLC7A7*-associated monogenic lupus

Characteristics	1	6	7	9	10	12
age at onset (Y)	10	3	5	6	6	8
age at diagnosis (Y)	10	3	10	6	6	8
onset syndrome	fever, anemia, thrombocytopenia	fever, anemia	fever, thrombocytopenia	fever	seizures, nephrotic syndrome	fever, limb gangrenes
SLEDAI at diagnosis	5	9	10	14	24	13
Constitutional symptom	fever	fever	fever	fever	-	fever, fatigue
Mucocutaneous involvement	-	-	-	-	-	limb gangrenes
Hematological involvement	+	+	+	+	-	+
anemia	+	+	+	+	-	+
leukopenia	+	-	+	-	-	-
thrombocytopenia	+	+	+	-	-	-
Coomb’s test	+	+	-	+	-	+
Renal involvement	+	+	+	+	+	-
proteinuria	mild	mild	mild	mild	massive	-
hematuria	-	-	+	-	+	-
renal biopsy	*NA	LN II	LN III	*NA	LN V	*NA
Central nervous system						
seizures	-	-	-	+	+	+
loss of consciousness	-	-	-	+	+	+
cerebral MRI	-	Cerebral atrophy	Abnormal signals in the basal ganglia region	Subdural hemorrhage Abnormal signals in the thalamus	Cerebral atrophy	-
EEG	EEG background activity slows down	EEG background activity slows down	-	EEG background activity slows down	Epileptic wave	spike and slow waves
Pulmonary involvement						
chest CT scan	Interstitial lung disease	Interstitial lung disease	Interstitial lung disease	alveolar proteinosis	-	Interstitial lung disease
pulmonary function test	*NA	mild obstructive ventilation dysfunction	mild obstructive ventilation dysfunction	mild obstructive ventilation dysfunction	-	-
DLCO (%)	*NA	*NA	73.8	*NA	-	-
KL-6 (u/L)	*NA	944	*NA	10,000	*NA	1623
Blood ammonia at onset	209	175	17	185	161	248
ferritin	> 2000	> 2000	1065	> 2000	527	> 2000
ANA	1:100	1:1280	1:100	1:1280	1:100	1:640
Anti-dsDNA	-	-	+	+	-	+
anti-Sm	-	-	-	-	-	-
anti-RNP/Sm	-	+	-	-	-	-
anti-SSA	-	+	+	+	+	+
anti-nucleosome antibody	-	-	+	+	-	-
anti-ribosomal p protein antibody	-	-	+	-	-	-
anti histone antibodies	-	-	+	+	-	-
other autoantibody	-	anti-Scl70, RF	PANCA	-	-	-
IgG (g/L)	18.4	24.8	19.92	18.54	17.2	33.2
CH50 (U/ml)	< 10	< 10	67	11	80	< 10
C3 (g/L)	0.36	0.38	1.32	0.33	1.41	0.24
C4 (g/L)	0.06	0.08	0.46	0.03	0.14	0.04
LA	1.3	1.55	*NA	*NA	*NA	0.99
anti-β2GP1(RU/ml)	21.4	125.2	*NA	*NA	*NA	110.4
ACA-IgG (U/ml)	< 2	2.8	*NA	-	-	5.8
ACA-IgM (U/ml)	22	2.9	*NA	-	-	3.4
ACA-IgA (U/ml)	< 2	3.2	*NA	-	-	< 2

*NA: Not analysis (test was not performed)

Abbreviations: SLE, systemic lupus erythematosus; SLEDAI, systemic lupus erythematosus disease activity index; LA, Lupus anticoagulant; EEG, electroencephalography; DLCO, Diffusing Capacity of the Lung for Carbon Monoxide; KL-6, Krebs von den Lungen-6

The significance of *SLC7A7* was further highlighted in our broader cohort. Among 171 childhood-onset SLE (cSLE) patients who underwent genetic testing, 19 were diagnosed with monogenic lupus. *SLC7A7* was the most frequently mutated gene in this group, responsible for 31.6% (6/19) of cases, establishing it as a principal genetic cause of monogenic lupus in our center. Furthermore, the splice-site mutation c.625 + 1G > A was the predominant allele, present in 6 of the 12 alleles among the six patients with *SLC7A7*-associated monogenic lupus, consistent with its high frequency in the LPI cohort (Fig. 2A-B).

### Clinical phenotype of *SLC7A7*-associated Monogenic lupus

Six patients (cases 1, 6, 7, 9, 10, and 12) were diagnosed with SLE (LPI + SLE+), representing 50% of the LPI cohort. All six LPI + SLE+ patients fulfilled both the 2012 SLICC and the 2019 EULAR/ACR classification criteria for SLE. These patients presented a distinct and severe clinical phenotype. Constitutionally, fever was a prominent presenting feature in five patients (83.3%). Hematological involvement was observed in five patients (83.3%), all of whom had anemia (5/5), alongside leukopenia (2/5) and thrombocytopenia (3/5).

Neurological and renal manifestations were paramount. Central nervous system (CNS) involvement was universal (6/6, 100%), with three patients experiencing overt seizures and the remainder showing subclinical abnormalities on imaging or electroencephalography. Renal involvement was equally universal. All six patients had evidence of renal tubulopathy, indicated by elevated urinary α1-microglobulin and N-acetyl-beta-D-glucosaminidase (NAG). Glomerular disease was also common: four patients had isolated proteinuria, while two presented with both proteinuria and hematuria. Kidney biopsies in three patients (Cases 6, 7, and 10) revealed class II, III, and V lupus nephritis, respectively. Immunofluorescence staining demonstrated pervasive immune complex deposits (IgA, IgG, IgM, C3, C4, C1q) (Fig. 2C).

The immunologic profile was characterized by a high frequency of specific autoantibodies. Anti-SSA/Ro antibodies were positive in all six patients (100%). In contrast, anti-dsDNA was detected in only three patients (50%), and anti-Sm was notably negative in five (83.3%) of the six patients. Hypocomplementemia (low C3 and/or C4) was observed in three patients.

### Distinct phenotype of *SLC7A7*-associated monogenic lupus (LPI + SLE+) compared to genetic-negative cSLE

**Fig. 3:**
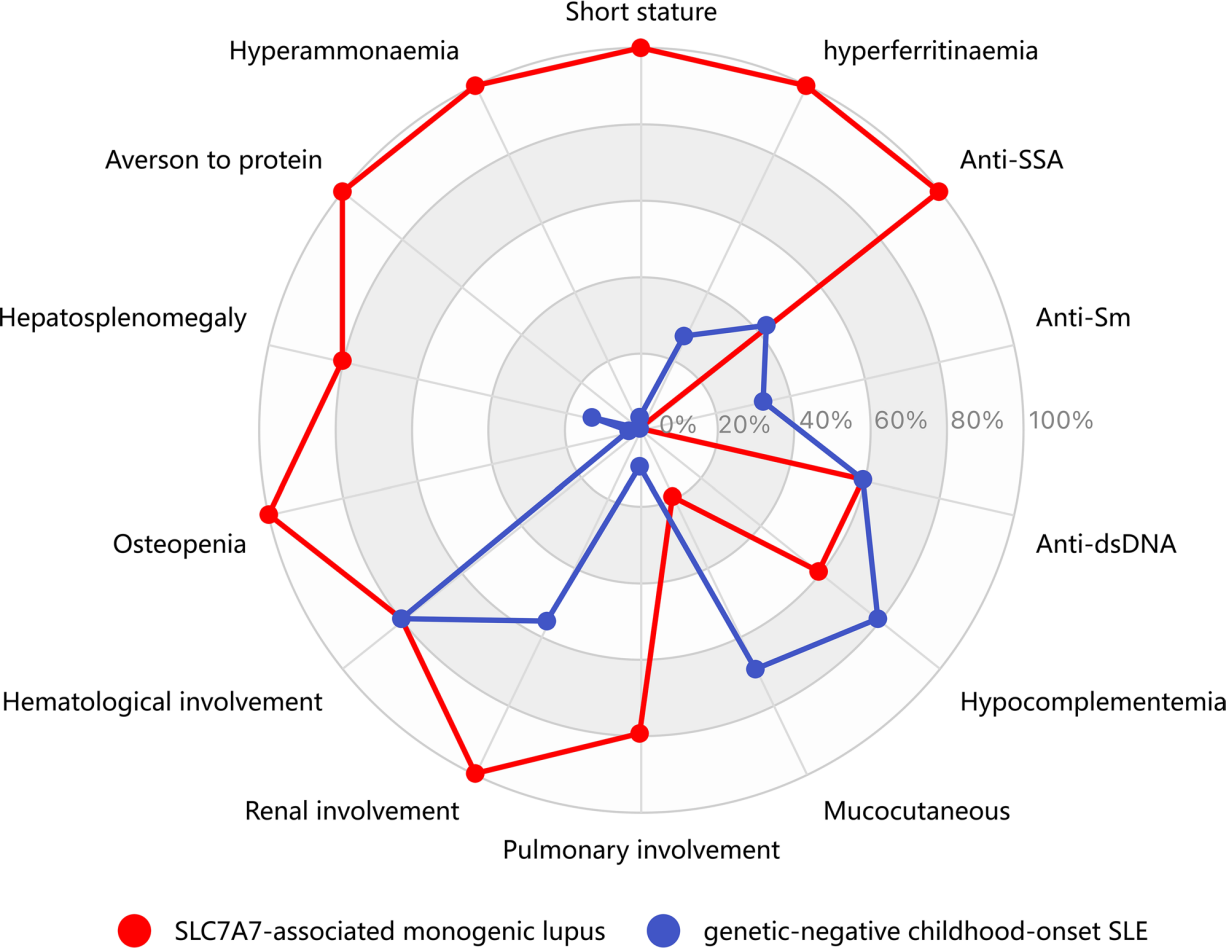
Distinctive phenotypic profile of *SLC7A7*-associated monogenic lupus compared to genetic-negative childhood-onset SLE. This radar plot contrasts the frequency of key clinical and laboratory features between two well-defined pediatric lupus subgroups: patients with *SLC7A7*-associated monogenic lupus (LPI + SLE+, *n* = 6, red contour) and those with genetic-negative childhood-onset SLE (*n* = 136, blue contour). Each radial axis represents a specific feature; the distance from the center indicates its relative frequency within the respective subgroup (0% at the center to 100% at the outermost ring). The *SLC7A7*-associated subgroup is distinguished by the universal presence (100% frequency) of a consistent clinical phenotype: the metabolic triad (aversion to protein, short stature, and osteopenia) accompanied by the laboratory findings of hyperammonemia and hyperferritinemia. These features are markedly less common or absent in the genetic-negative cSLE group (0%, 3%, 3%, 0%, and 27%, respectively; all *p* < 0.05). Line color and contour differentiate the two subgroups

We compared the six LPI + SLE+ patients with 136 genetic-negative cSLE patients (Table 2; Fig. 3). The LPI + SLE+ group was diagnosed with SLE at a significantly younger age.

**Table 2:** Key differentiating characteristics between *SLC7A7-*associated monogenic lupus and genetic-negative childhood-onset SLE

Characteristics	SLC7A7-associated monogenic lupus	Gene-negative cSLE		*P* value	
number	6	136			
Sex (female: male)	3:2	96:40	0.634		
Age at diagnosis	6.6 ± 2.6	10.5 (8.0, 12.5)	**0.021**		
Protein intolerance	6 (100%)	0 (0%)		**0.000**	
Short stature at onset	6 (100%)	4 (3.7%)	**0.000**		
BMD (z score)	-3.1 ± 0.65	0.031 ± 0.98	**0.000**		
SLEDAI at diagnosis of SLE	13 (10, 19)	12 (6, 20)	0.642		
Fever	5(83.3%)	63(46.3%)	0.191		
Mucocutaneous	1 (16.7%)	97 (71.3%)	**0.030**		
Arthritis/arthralgia	0 (0%)	42 (30.9%)	0.325		
Hematological involvement	5 (83.3%)	109 (80.1%)	1.000		
Renal involvement	6 (100%)	76 (55.9%)	0.134		
Pulmonary involvement	5 (83.3%)	13 (9.6%)	**0.001**		
Central nervous system	5 (83.3%)	17 (12.5%)	**0.002**		
Hepatosplenomegaly	5 (83.3%)	18 (13.2%)	**0.002**		
Ferritin	2000 (796.0, 2000)	250.9 (122.3, 521.7)	**0.002**		
Lactate dehydrogenase (U/L)	685.0 (566.5, 6413.5)	297.5 (238.8, 399.3)	**0.001**		
IgG (g/L)	22.73 ± 6.52	17.94 ± 7.52	0.153		
C3 (g/L)	0.38 (0.29, 1.37)	0.57 (0.35, 0.90)	0.867		
C4 (g/L)	0.08 (0.04, 0.30)	0.07 (0.03, 0.18)	0.659		
CH50 (U/ml)	11 (10, 73.5)	10 (10, 29.25)	0.900		
Anti-dsDNA	3 (50.0%)	81 (59.6%)	1.000		
Anti-Sm	0 (0%)	45 (33.1%)	0.284		
Anti-SSA	5 (83.3%)	59 (43.4%)	**0.041**		

The characteristic metabolic triad of LPI—protein intolerance, short stature, and osteopenia—was universally present in the LPI + SLE+ group (100% for all three features) but absent or rare in the genetic-negative cSLE group (protein intolerance: 0/136, 0%; short stature: 5/136, 3.7%; osteopenia: 4/126, 3.2%). These differences were statistically significant (*p* < 0.001).

Beyond this distinctive metabolic profile, the phenotypic patterns further diverged. The LPI + SLE+ group had significantly lower rates of mucocutaneous manifestations (16.7% vs. 71.3%, *p* = 0.030) but dramatically higher rates of lung involvement (83.3% vs. 5.1%, *p* < 0.001), central nervous system involvement (100% vs. 19.1%, *p* < 0.001), and hepatosplenomegaly (83.3% vs. 13.2%, *p* = 0.002).

Immunologically, there were no significant differences in complement or specific autoantibody levels. However, the LPI + SLE+ group exhibited pronounced elevations in ferritin and lactate dehydrogenase (*p* = 0.002 and *p* = 0.001, respectively) (Table 2; Fig. 3).

Post hoc power analyses using G*Power (version 3.1), with α = 0.05 and effect sizes derived from the observed differences, indicated that the statistical power for key comparisons (protein intolerance, short stature, and low bone mineral density) was 1.00, confirming adequate power despite the small sample size.

### Treatment and follow-up of LPI + SLE+ patients

All LPI + SLE+ patients received a combined regimen of metabolic therapy and immunosuppression. Metabolic support included a low-protein diet, supplementation with citrulline, arginine, and lysine, and management of hyperammonemia episodes. For SLE, all patients were treated with hydroxychloroquine. Pulse methylprednisolone was administered to cases 1, 6, 9, and 10 for indications including secondary HLH, neuropsychiatric lupus, and severe lupus nephritis. Most patients required additional immunosuppressants, with one patient receiving belimumab.

Over a mean follow-up of 5.5 years (range: 0.5–13 years), one patient (Case 1) died from severe COVID-19. The remaining five patients all achieved Lupus Low Disease Activity State (LLDAS). The time to LLDAS for cases 6, 7, 9, 10, and 12 was 1 year, 2.5 years, 0.5 years, 1 year, and 0.5 years, respectively. Three of these patients successfully discontinued glucocorticoids after 3–5 years. Following disease control, three patients received growth hormone therapy, with two achieving normal height. While no patient developed chronic kidney dysfunction, elevated urinary NAG levels persisted in several patients, suggesting ongoing subclinical tubular injury.

## Discussion

Since the first description of LPI, considerable clinical heterogeneity has been observed among patients worldwide. Common clinical manifestations include protein aversion, failure to thrive, hyperammonemia, hepatosplenomegaly, osteoporosis, alveolar proteinosis, and immunological disorders. While LPI is more frequently reported in Finland, Italy, France, and Northern Japan, the first case in China was reported by the Neurology Department of Huashan Hospital in 2016. To date, including the 12 cases from our center, a total of 23 cases have been documented in China. Notably, these Chinese cases were reported by diverse clinical departments, underscoring the need for heightened awareness among pediatricians to facilitate early diagnosis. We compared the clinical manifestation and organ involvement described in studies from other countries with those identified in China [12, 15–22, 27–31].

The clinical presentation of LPI in China shares similarities with global reports, including gastrointestinal, metabolic, and musculoskeletal involvement. However, several distinctive features emerged. Osteopenia was universally present in reported Chinese cases (16/16, 100%), a proportion markedly higher than the 37% (59/157) cited in a 2021 international review [13]. While renal tubulopathy was universal (15/15, 100%) in Chinese reported LPI patients, no cases of progressive kidney impairment (0/15, 0%) have been documented, contrasting with high rates of renal dysfunction in Finnish (25/41, 61.0%) and French (6/16, 37.5%) cohorts [30, 32]. This discrepancy may be partly attributable to the younger age of the Chinese cohort and shorter follow-up, as renal impairment in LPI typically manifests in adulthood. It is imperative to extend the follow-up duration to thoroughly monitor and assess renal impairment in this cohort of pediatric LPI patients.

**Fig. 4:**
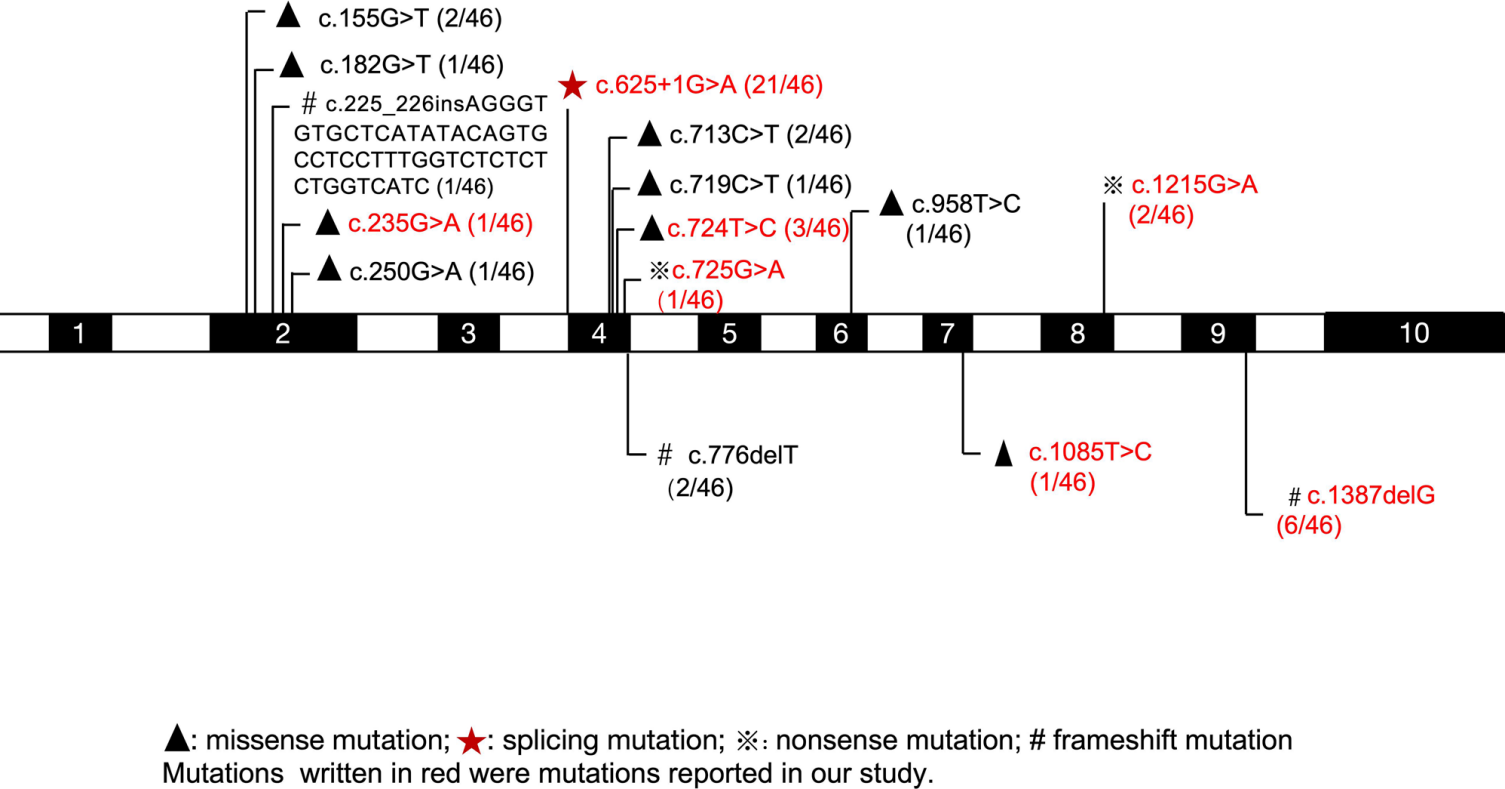
Mutational landscape of *SLC7A7* in Chinese patients with lysinuric protein intolerance. All documented pathogenic variants identified in this cohort and previously reported in Chinese patients are plotted on the protein schematic. The recurrent c.625 + 1G > A mutation and a cluster of variants in exon 4 define a potential mutational hotspot in this population

The LPI gene, *SLC7A7*, was identified in 1999 through a positional candidate cloning approach [33, 34]. Genetically, nearly 80 mutations in *SLC7A7* have been reported globally without a universal hotspot, with regional founder effects observed in Finland (c.895–2 A > T) and the northern part of Japan (p.R410X) [35]. Our genetic analysis, combined with a review of previously reported cases in China [15–22], reveals a distinct mutational spectrum for LPI in the Chinese population. In our study, we identified eight mutations, among which the nonsense mutation c.725G > A (p.W242X) is a novel mutation. The splice-site mutation c.625 + 1G > A was the most prevalent allele (58.3%, 14/24). This finding is corroborated by the national reported data, where c.625 + 1G > A constitutes the majority of reported mutant alleles (43.5%, 20/46 alleles) to date (Table S2, Fig. 4). The splice-site mutation c.625 + 1G > A was initially identified in a Japanese LPI patient, with a frequency of 0.11 reported among Japanese LPI patients [29, 36]. Furthermore, the majority of *SLC7A7* mutations in Chinese patients are located in exon 4 (65.2%, 30/46 alleles), suggesting a potential mutational hotspot. This predominant mutation profile may have implications for genetic screening and diagnosis strategies for suspected LPI in China. Collectively, these findings raise the possibility that c.625 + 1G > A may represent a mutational hotspot specific to the Chinese LPI population, a hypothesis that warrants confirmation through the genetic profiling of future cases. However, no clear genotype-phenotype correlation was identified, as patients with identical genotypes exhibited divergent prognoses and organ involvement.

Immunological dysregulation emerged as a predominant feature in our LPI cohort, affecting 58.3% (7/12) of patients. Notably, six patients were diagnosed with SLE, representing 50.0% (6/12) of our cohort. Among these six SLE patients, two concurrently developed HLH. Additionally, one patient presented with HLH alone. This pattern of frequent immunological involvement is consistent at the national level. Among all reported LPI cases in China (including ours), 43.5% (10/23) of patients developed immunological disorders. These include eight cases of SLE (8/23, 34.8%), comprising five with SLE alone and three with SLE and HLH, alongside one case of isolated HLH and one case of immune thrombocytopenia [15, 20, 21]. The prevalence of SLE in Chinese LPI patients (34.8%) significantly exceeds rates documented in other populations: France (6.3%), Finland (3.4%), and a 2021 systematic review (3%) [12, 13, 30]. This striking disparity suggests that immunological dysfunction, particularly SLE, may represent a prominent feature of LPI in Chinese patients, especially those seen at tertiary referral centers. Consequently, we emphasize that management of LPI patients must include rigorous and proactive immunological surveillance, extending beyond metabolic follow-up alone.

The pathogenesis of SLE is complex, encompassing abnormalities across all aspects of the immune system. Central to SLE pathogenesis is a breakdown in B-cell tolerance to nucleic acids and the activation of the type I interferon (IFN) pathway and complement. Most gene variants implicated in monogenic lupus relate to these interconnected processes, including complement deficiency, activation of the type I IFN pathway, and breakdown of B-cell and T-cell tolerance. It is generally believed that metabolic diseases account for only a small portion of monogenic lupus; however, in our center, the *SLC7A7* gene is currently the most common pathogenic gene identified, accounting for 31.6% (6/19) of the monogenic lupus cases.

As the most prevalent monogenic lupus gene in our centre, *SLC7A7* mutations define a distinct clinical subset in this cohort. Patients with *SLC7A7*-associated monogenic lupus presented at a younger age and exhibited more frequent central nervous system involvement than those with gene-negative SLE. The increased frequency of neuropsychiatric symptoms may be partly attributable to hyperammonemia, a common feature of LPI, though ammonia levels were not systematically assessed at SLE onset in our cohort. The clinical characteristics of *SLC7A7*-associated monogenic lupus mutations differ significantly from those of gene-negative cSLE, often reflecting the metabolic presentations associated with LPI. The universal presence of the metabolic triad—protein intolerance, short stature, and osteopenia—in all LPI + SLE+ patients, contrasted with its near absence in genetic-negative cSLE, provides a clear clinical signature that should prompt consideration of underlying LPI and *SLC7A7* genetic testing in children presenting with SLE, particularly when accompanied by hyperammonemia or other metabolic abnormalities. This approach facilitates the rapid identification of *SLC7A7*-associated monogenic lupus, enabling early diagnosis.

The mechanism linking *SLC7A7* mutations to SLE development remains incompletely elucidated, but accumulating experimental evidence points to macrophage dysfunction as a potential contributor.Barilli’s study demonstrate that *SLC7A7* deficiency impairs y + L system transporter activity, reducing L-arginine efflux in immune cells [37]. This gene expression is upregulated during macrophage differentiation induced by various stimuli including PMA, VD3, and ATRA, primarily through PKCβ, MAPK, and NF-κB signaling pathways [38]. Rotoli et al. reported in 2018 that siRNA-mediated silencing of SLC7A7/y⁺LAT1 in human THP-1 cells and A549 airway epithelial cells resulted in a significant increase in the expression and release of the inflammatory mediators interleukin-1β (IL-1β) and tumor necrosis factor-α (TNF-α). These effects were independent of intracellular arginine levels and were primarily regulated at the transcriptional level through activation of the NF-κB signaling pathway. This led to the hypothesis that SLC7A7 may possess an anti-inflammatory function beyond its role in amino acid transport [39]. These findings collectively suggest that *SLC7A7* deficiency disrupts immune homeostasis through multiple mechanisms, though the precise pathway to SLE pathogenesis requires further investigation.

At our center, all patients with *SLC7A7*-associated monogenic lupus received both immunosuppressive therapy and metabolic therapy. Notably, 5 of 6 LPI + SLE+ patients (83.3%) achieved LLDAS and maintained it until the last follow-up. To our knowledge, approximately 10 cases of LPI combined with SLE have been reported [13, 14, 17, 20, 30, 40–42]. Except for one case, which lacked detailed clinical features, all remaining cases exhibited renal involvement characterized by hematuria and/or varying degrees of proteinuria, ranging from mild to severe. Among these patients, five died, with four of the deceased patients being reported prior to 2000. Notably, all cases of LPI complicated by SLE reported after 2000 received immunosuppressive therapy and metabolic therapy. The immunosuppressive treatments included cyclophosphamide, azathioprine, mycophenolate mofetil, and even one patient underwent hematopoietic stem cell transplantation. Early diagnosis, along with prompt immunotherapy and metabolic treatment, has been shown to lead to improved prognoses.

This study has several limitations that should be acknowledged. First, as a retrospective single-center study, it is subject to potential referral bias and reliance on historical medical records, which may lack systematic data collection. Second, a significant limitation is the small sample size of our LPI cohort, which inherently affects the statistical robustness of our findings. The limited sample size increases the risk of type II errors for more subtle associations and may lead to less precise effect size estimates. Third, a diagnostic limitation in our study relates to the significant delay in LPI diagnosis within the Chinese healthcare context. The median diagnostic delay of 7 years from symptom onset suggests that many patients with milder clinical presentations may remain undiagnosed or be misdiagnosed in primary care settings. This diagnostic gap likely results from both limited patient awareness leading to under-utilization of specialized medical services, and insufficient physician recognition of this rare disease’s diverse manifestations. Consequently, our cohort from a tertiary referral center may represent a selected population with more severe or advanced disease, potentially explaining the notably high proportion of immunological abnormalities observed in our study. Future multi-center collaborative studies with larger sample sizes and longer follow-up durations are warranted to validate and extend our observations.

In summary, our study delineates a described profile of LPI in China, characterized by a unique genetic spectrum with c.625 + 1G > A as the predominant mutation, a remarkably high prevalence of SLE in this cohort, and universal metabolic abnormalities. The metabolic triad may serve as a valuable clinical marker for suspecting SLC7A7-associated monogenic lupus. These findings suggest the importance of combined metabolic and immunosuppressive therapy, and point to the need for heightened immunological surveillance in Chinese LPI patients.

## Conclusion

In our cohort, *SLC7A7* was the most frequently identified gene among the monogenic lupus-related genes investigated. The c.625 + 1G > A variant was the most commonly observed mutation in this patient group. Notably, within our cohort, patients with LPI appeared to have a relatively high occurrence of SLE, highlighting the need for enhanced immunological surveillance during follow-up. The consistent presence of the metabolic triad—protein intolerance, short stature, and osteopenia—in our *SLC7A7*-associated monogenic lupus cases may represent a clinically informative pattern that could warrant consideration of genetic testing for *SLC7A7*. Our preliminary experience suggests that patients with *SLC7A7*-associated monogenic lupus may benefit from combined therapy addressing both immunological and metabolic aspects of the disease. Given the small sample size, these observations should be interpreted cautiously; nevertheless, timely recognition and combined management in this cohort were accompanied by favorable clinical courses, with most patients achieving and maintaining low disease activity.

## Supplementary Information

Below is the link to the electronic supplementary material.

Supplementary Material 1

## Data Availability

All data generated or analysed during this study are included in this published article and its supplementary information files.

## Abbreviations

CAAs: Cationic Amino Acids
CNS: Central nervous system
cSLE: Childhood-onset Systemic Lupus Erythematosus
IFN: Interferon
IL-1β: Interleukin-1β
HLH: Haemophagocytic Lymphohistiocytosis
IQR: Interquartile Range
LLDAS: Lupus Low Disease Activity State
LPI: Lysinuric Protein Intolerance
NAG: N-acetyl-beta-D-glucosaminidase
SD: Standard Deviation
SLE: Systemic Lupus Erythematosus
TNF-α: Tumor Necrosis Factor-α
y+LAT1: y + L amino acid transporter-1
4F2hc: Surface antigen 4F2 heavy chain
