# Supplementary material for: Monogenic lupus with SLC7A7 mutations: a retrospective study from a Chinese center
Source: Orphanet J Rare Dis. 2026 Mar 13;21:161. doi: 10.1186/s13023-026-04258-w (PMC13097903; doi:10.1186/s13023-026-04258-w)
Supplement: Supplementary file 1 — Supplementary Material 1 [file 13023_2026_4258_MOESM1_ESM.docx]

Supplement

Table S1 Clinical and laboratory features of patients with *SLC7A7* mutation

|  | 1* | 2* | 3 | 4 | 5** | 6** | 7*** | 8*** | 9 | 10 | 11 | 12 |
| --- | --- | --- | --- | --- | --- | --- | --- | --- | --- | --- | --- | --- |
| Year of birth | 2008 | 2007 | 2015 | 2010 | 2012 | 2017 | 2006 | 2011 | 2010 | 2005 | 2007 | 2015 |
| Gender | female | female | female | female | male | female | female | male | female | male | male | male |
| age at onset of LPI  (years) | 1 | 1.00 | 1 | newborn | newborn | 2 | 0.3 | 3 | 0.5 | 6 | 6 | 1 |
| Age at diagnosis of  LPI (years) | 8 | 9 | 2 | 5 | 8 | 3 | 10 | 6 | 6 | 6 | 15 | 8 |
| Age at diagnosis of  HLH (years) | 8 | / | / | / | / | 3 | / | / | / | / | / | / |
| Age at diagnosis of  SLE (years) | 10 | / | / | / | / | 3 | 10 | / | 6 | 11 | / | 8 |
| Height at diagnosis | <P3rd | <P3rd | <P3rd | <P3rd | <P3rd | <P3rd | <P3rd | <P3rd | <P3rd | <P3rd | <P3rd | <P3rd |
| Gastrointestinal symptoms | vomiting | vomiting,  diarrhea | vomiting | vomiting | vomiting,  diarrhea | - | diarrhea,  hematochezia | - | vomiting | - | - | - |
| protein-rich food aversion | + | + | + | + | + | + | + | + | + | + | + | + |
| failure to thrive | + | + | + | + | + | + | + | + | + | + | + | + |
| short stature | + | + | + | + | + | + | + | + | + | + | + | + |
| hyperammonaemia | 209umol/L | 75umol/L | 292.3umol/  L | 215umol/L | 240umol/L | 128umol/L | 115umol/L | 188umol/L | 178uml/L | 240umol/L | 548umol/L | 248umol/L |
| Hepatosplenomegaly | Hepatomegaly, splenomegaly | Hepatomegaly, splenomegaly | Hepatomegaly, splenomegaly | Hepatomegaly, splenomegaly | Hepatomegaly, splenomegaly | Hepatomegaly | - | Hepatomegaly | Hepatomegaly, splenomegaly | Hepatomegaly | - | Hepatomegaly, splenomegaly |
| Elevated transaminase | + | + | + | + | + | + | - | + | + | + | - | - |
| Hematological involvement | pancytopenia | anemia, leukopenia | - | anemia | - | anemia,  thrombocytopenia | pancytopenia | - | anemia | anemia | - | anemia |
| Cognitive impairment | + | - | - | - | + | - | - | - | + | + | + | - |

| Seizures | - | - | - | - | - | - | - | - | + | + | + | + |
| --- | --- | --- | --- | --- | --- | --- | --- | --- | --- | --- | --- | --- |
| Coma | - | - | - | - | - | + | - | - | - | + | + | + |
| Renal involvement | mild  proteinuria | elavated of  NAG | elevated of  NAG and  a1MU | mild  proteinuria | elevated of  NAG and  a1MU | mild  proteinuria | mild  proteinuria | elevated of  NAG | elevated of  NAG | nephrotic  syndrome | elevated of  NAG | elevated of  NAG |
| eGFR < 90 ml/min/1.73m2 | - | - | - | - | - | - | - | - | - | - | - | - |
| Hypercalciuria | NA | NA | NA | + | NA | NA | NA | NA | - | + | NA | - |
| Bone mineral density | -4.2 | -3.6  fracture | -2.48 | -4.1 | NA | -3 | -2.8 | -3.5 | -3.3 | -3 | -2.6 | -2.2 |
| Pulmonary involvement | Interstitial lung diease | Interstitial lung disease | - | Alveolar proteinosis | - | Interstitial  lung disease | Interstitial lung disease | - | Alveolar proteinosis | - | - | Interstitial lung disease |
| Ferritin (ng/ml) | >2000 | >2000 | >2000 | >2000 | >2000 | >2000 | 1471 | 419.6ng/ml | >  2000ng/ml | 527 | 679.1 | >2000 |
| LDH (U/L) | 3315 | 1023 | 1486 | 1006 | 2197 | 11450 | 1377 | 731 | 939 | 604 | 362 | 529 |
| *SLC7A7* mutations | c.1215G>A  (p.W405X)  /c.1387delG (p.V463Cfs*56) | c.1215G>A  (p.W405X)  /c.1387delG (p.V463Cfs*56) | c.625+1G>A/  c.625+1G>A | c.625+1G>A/  c.625+1G>A | c.625+1G>A/  c.625+1G>A | c.625+1G>A/  c.625+1G>A | c.625+1G>A/  c.625+1G>A | c.625+1G>A/  c.625+1G>A | c.625+1G>A/  c.235G>A (p.G79R) | c.625+1G>A/  c.1085T>C# (p.L362P) | c.725G>A# (p.W242X)/  c.1387delG (p.V463Cfs*56) | c.724T>C (p.W242R)/  c.724T>C (p.W242R) |

*,** and *** refer to 3 different families. Cases without asterisk are from different families

Abbreviations: SLE, systemic lupus erythematosus; LPI, lysinuric protein intolerance; HLH, haemophagocytic lymphohistiocytosis; LDH, lactate dehydrogenase; eGFR, estimated Glomerular Filtration Rate; NA, not available;NAG, N-acetyl-beta-D-glucoaminidase; a1MU, α1microglobulinuria.

Table S2 *SLC7A7* mutations reported in Chinese LPI patients

| cases | Sisters from one families^16^ | 114 | 120 | 119 | 2 sisters17 | 117 | 1^15^ | 118 | 121 | 2 sisters22 |
| --- | --- | --- | --- | --- | --- | --- | --- | --- | --- | --- |
| *SLC7A7* mutation 1 | c.1387delC | c.625+1G>A | c.625+1G>A | c.713C>T | c.1215G>A  (p.W405X) | c.625+1G>A | c.1387delG | c.724T>C  (p. W242R) | c.625+1G>A | c.776delT  (p.L259Rfs*18) |
| *SLC7A7* mutation 2 | IVS4+ 1C>T | c.182G>T  (p.G61V) | c.250G>A  (p.V84I) | c.713C>T | c.1387delG  (p.V463Cfs*56) | c.625+1G>A | c.958T>C | c.719C>T  (p. S240L) | p.W76Rfs*110 | c.155G>T  (p.G52V) |

* The two sisters were case 1 and case 2 in our study.
